# Effects of Four-Week Supplementation with a Multi-Vitamin/Mineral Preparation on Mood and Blood Biomarkers in Young Adults: A Randomised, Double-Blind, Placebo-Controlled Trial

**DOI:** 10.3390/nu7115451

**Published:** 2015-10-30

**Authors:** David J. White, Katherine H. M. Cox, Riccarda Peters, Andrew Pipingas, Andrew B. Scholey

**Affiliations:** Centre for Human Psychopharmacology, Swinburne University of Technology, Hawthorn, VIC 3122, Australia; dawhite@swin.edu.au (D.J.W.); katehmcox@gmail.com (K.H.M.C.); riccardapeters@swin.edu.au (R.P.); apipingas@swin.edu.au (A.P.)

**Keywords:** multivitamin, mineral, B-vitamins, micronutrient, mood, homocysteine

## Abstract

This study explored the effects of four-week multi-vitamin and mineral (MVM) supplementation on mood and neurocognitive function in healthy, young adults. Fifty-eight healthy adults, 18–40 years of age (*M* = 25.82 years, SD = 4.87) participated in this randomised, double-blind, placebo-controlled trial, in which mood and blood biomarkers were assessed at baseline and after four weeks of supplementation. Compared to placebo, MVM supplementation was associated with significantly lowered homocysteine and increased blood B-vitamin levels (*p* < 0.01). MVM treatment was also associated with significantly improved mood, as measured by reduced scores on the “depression-dejection” subscale of the Profile of Mood States (*p* = 0.018). These findings suggest that the four weeks of MVM supplementation may have beneficial effects on mood, underpinned by elevated B-vitamins and lowered homocysteine in healthy young adults.

## 1. Introduction

B-vitamins play a key role in a host of physiological processes critical to human health. In maintaining brain function, these micronutrients are involved in the synthesis of neurotransmitters (as well as proteins, lipids and hormones), modulation of receptor binding and glucose metabolism. In one of the many important roles of these micronutrients as cofactors in the one-carbon metabolism pathway, vitamins B6, B9 (folic acid) and B12 also reduce homocysteine to methionine and glutathione [1].

Given the prominent role of B-vitamins in a range of critical physiological processes *in vivo*, it follows that deficiency, often linked with elevated homocysteine, has been associated with a range of negative neurocognitive and psychiatric consequences in older adults [2,3,4,5,6,7,8,9,10,11]. Despite this, randomised controlled intervention trials targeting deficiency in a single B-vitamin, or a small subset, have produced largely null findings in terms of reducing the risk of cognitive decline or Alzheimer’s disease in at-risk aging samples (reviewed in [12,13,14]). Among a number of factors likely contributing to these null findings is the emerging understanding of genetic polymorphisms contributing to variability in B-vitamin-related processes [15] and the notion that correcting a single micronutrient deficiency assumes adequate availability of other micronutrients also involved in these complex biochemical pathways [16].

An alternative intervention strategy has been to explore combination multivitamin/mineral (MVM) treatments, which include high doses of B-vitamins in addition to other micronutrients. This research has typically studied populations with no diagnosed clinical deficiency, instead operating on the principle that optimal micronutrient levels are not simply those that are required to prevent clinical deficiency. Whilst developed nations show relatively low prevalence of clinical micronutrient deficiency among adults, definitions of deficiency are based on the prevention of specific physical illness related to deficiency in a specific micronutrient, and as such, this perspective does not inform optimal micronutrient intake [17]. Supporting the distinction between clinical deficiency and sub-optimal micronutrient status [18,19], studies exploring MVM administration in healthy adults have reported potential benefits for cognitive function and mood.

Benefits for subjective stress and mood in particular have been observed in healthy adults as a result of MVM supplementation (for a meta-analytic review, see [20]). Initial evidence of benefits for mood after MVM supplementation in healthy adults was described over 12 months of supplementation [21]. Subsequently, self-report measures of anxiety and stress were found to be reduced following 28 days of MVM supplementation in a sample of 80 healthy males [22], with similar benefits reported for well-being, anxiety and stress after 30 days of supplementation in a sample of 300 participants exhibiting high levels of stress at baseline [23]. Sixteen weeks of MVM supplementation with additional herbal ingredients were found to improve subjective mood in a healthy adult sample using both qualitative [24] and quantitative assessments from at-home mobile phone assessments of stress, anxiety and fatigue, though not during laboratory assessment [25]. A trial exploring 33 days of MVM supplementation was shown to benefit mood and reduce stress, as well as self-reported general health in a sample of 215 males aged 30–55 [26]. Stough *et al.* [27] reported reductions in personal strain as measured by the Occupational Stress Inventory, in addition to reduced depression-dejection and confusion ratings on the Profile of Mood States after 90 days of MVM supplementation in healthy adults. Recent evidence suggests that MVM supplementation may also reduce stress and anxiety in the period following a natural disaster [28,29,30].

Studies exploring changes in cognitive performance associated with MVM supplementation have observed a less consistent pattern of outcomes [31]. Positive effects on aspects of cognitive function, specifically immediate free recall, have been noted using meta-analytic methods with this research literature [32]; however, a number of cognitive domains were found to be under-researched. Of those to study pre-midlife healthy adults, the study of Kennedy *et al.* [26] reported improved performance on serial three and serial seven subtraction tasks after MVM treatment in addition to the mood benefits described above in adult males (aged 30–55) following 28 days of supplementation. In younger adult females, 63 days of MVM supplementation were associated with improved cognitive performance on a multi-tasking paradigm, with a concomitant reduction in subjective fatigue associated with task completion [33]. As part of the 16-week trial MVM supplementation with additional herbal ingredients, Pipingas *et al.* [34] reported limited evidence of cognitive changes associated with MVM supplementation. Improvements in performance on the Stroop task were noted within the male sample; however, this trend failed to reach significance when correcting for multiple comparisons. Within this male sample receiving MVM treatment, the trend towards changing Stroop performance was correlated with the change in blood levels of vitamin B6 [34].

A significant proportion of the population report the use of MVM supplements across Australia, the United States and Europe [35,36,37]. In addition, there is increasing evidence that MVM supplementation may improve aspects of mood and possibly cognitive function, even in cognitively-intact, young, healthy adults. For these reasons, it remains important to further characterise the behavioural effects of MVM supplementation, including examining if the effects emerge earlier in a supplementation regimen and in younger populations than previously examined (in this case, following 28-day supplementation in young adults aged <40 years). We hypothesised that vitamin supplementation would improve mood as evaluated using the Profile of Mood States and would result in increased B-vitamins and reduced homocysteine. A further aim was to evaluate if the protection against the negative effects of workload stress observed in women who were supplemented with MVM for nine weeks [33] was evident following a shorter duration of supplementation.

## 2. Methods

This study followed a randomised, placebo-controlled, double-blind parallel groups design investigating the effects of 4 weeks of supplementation with a multi-vitamin and mineral preparation on mood, cognitive function and blood biochemical measures. The primary outcomes of this investigation were measures of functional brain activity, as measured using functional magnetic resonance imaging (fMRI) and steady state topography (SST), the outcomes of which will be reported elsewhere. The study was approved by the Swinburne University Human Research Ethics Committee (Ref SUHREC 2012/164) and was registered with the Australian New Zealand Clinical Trials Registry (ACTRN12612001043820).

### 2.1. Participants

**Figure 1:**
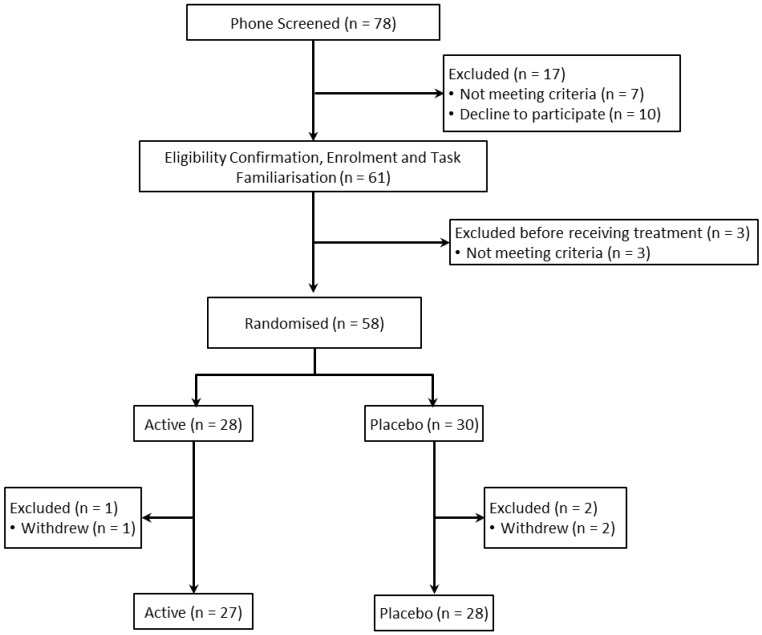
Flow of participants through the trial.

A total of 58 healthy young adults aged 18–39 years (M = 25.82, SD = 4.87) were enrolled in the study, with 55 completing the follow-up assessment. Participants were recruited via local media advertising. A history or current diagnosis of psychiatric disorder, heart disease, high blood pressure, diabetes or any condition that may interfere with normal food metabolism (for example, relevant food allergies or irritable bowel syndrome) were considered criteria for exclusion. Participants were also required to not be taking medication, herbal extracts, vitamin supplements or illicit drugs within 4 weeks prior to (and for the duration of) the study, with the exception of contraceptive pills or routine medications to treat benign conditions. The flow of participants through the trial is summarized in Figure 1 below.

### 2.2. Procedure

Participants attended the Centre for Human Psychopharmacology, at Swinburne University, on three occasions. After initial telephone screening, the first of these visits involved first obtaining informed consent, assessment of eligibility, demographic and morphometric information and familiarisation with the testing procedures. Following this visit, participants attended two identical morning assessment visits at baseline and following 28 days of supplementation with multivitamin/mineral or placebo, where the time of testing was held constant for each participant (starting 09:00 a.m. or 10:00 a.m.). Participants fasted and avoided any caffeinated products from 12 h prior to these testing sessions; in addition, participants refrained from drinking alcohol for 24 h before testing sessions. Upon arrival, participants had a fasting blood sample taken, after which a standardized breakfast was provided. Twenty minutes after breakfast and prior to undergoing neuroimaging [38] (the outcomes of functional brain activity assessments are to be reported elsewhere), participants completed a series of self-report mood questionnaires.

### 2.3. Treatments and Randomisation

Randomisation was conducted by a disinterested third party, whereby stratified randomisation was used to balance treatment assignment and representation of genders within each testing arm. Within these strata, randomisation numbers were assigned in ascending numerical order according to the date of randomisation.

Active and placebo treatments were in the form of effervescent tablets matched for colour and flavour, prepared by Bayer AG (Basel, Switzerland). Participants were instructed to take one tablet daily with breakfast, dissolved in at least 200 mL of water. Participants were provided with 30 tablets in case of minor delays in follow-up visit scheduling, but returned after 28 days of supplementation having not taken a treatment on the day of their return visit (to eliminate the influence of acute-on-chronic effects). Compliance was determined by the count of returned treatments. The active MVM is available over the counter as Berocca^®^ Performance, and the doses of nutrients are detailed in Table 1.

**Table 1:** Doses of vitamins and minerals in active multi-vitamin/mineral (MVM) treatment.

Nutrient	Amount
Vitamin C	500 mg
Thiamine monophosphoric acid ester chloride	18.54 mg
Riboflavin (vitamin B2)	15 mg
Nicotinamide (B3/niacin)	50 mg
Vitamin B5	23 mg
Vitamin B6	10 mg
Vitamin B12	0.01 mg
Folic acid (vitamin B9)	0.4 mg
Biotin (vitamin B7)	0.15 mg
Calcium	100 mg
Magnesium	100 mg
Zinc	10 mg

### 2.4. Blood Biochemical Assessment

At the beginning of each assessment session, venous blood was extracted via venipuncture of the antecubital fossa. Blood was collected into three tubes: a serum separator tube (8.5 mL); allowed to clot before centrifugation at 4000 rpm, analysed for homocysteine, vitamin B12, lipids, electrolytes, glucose, C-reactive protein (high sensitivity test) and indicators of liver and renal function; a lithium heparin tube (4 mL) was immediately wrapped in foil; analysed for vitamin B6 and an EDTA tube (4 mL), stored at room temperature prior to couriering; and analysed for red blood cell folate. Samples were couriered to a commercial pathology laboratory daily, where analysis was conducted.

### 2.5. Mood Assessment

Two aspects of mood were compared between the placebo and MVM groups. Recent mood was evaluated using the Profile of Mood States (POMS) and Perceived Stress Scale. SST imaging involves undergoing a continuous performance task [39] and a spatial working memory task [40] while wearing goggles that generate a 13-Hz flicker in the peripheral visual field. As such, SST is cognitively demanding and generates mental workload stress. Thus, mood changes during this cognitive challenge were used to index stress reactivity in the subset of 40 participants who underwent SST (measured using visual analogue mood scales and the state scale of the State-Trait Anxiety Inventory before and after the SST assessment).

#### 2.5.1. Profile of Mood States

The POMS [41] is an established 65-item self-report measure of mood across six sub-scales, described as “tension-anxiety”, “confusion-bewilderment”, “vigour-activity”, “anger-hostility”, “depression-dejection” and “fatigue-inertia”. Participants indicated on a 5-point scale from 0 (not at all) to 4 (extremely) the degree to which each of 65 mood-related adjectives describe feelings over the past week. Higher scores on each subscale indicate a greater presence of the mood factor, with a total mood disturbance (TMD) score computed as the sum of the five negative mood factors minus vigour-activity.

#### 2.5.2. Perceived Stress Scale

The Perceived Stress Scale-10 (PSS-10) [42] assesses the extent to which life events have been appraised as stressful over the past month. The scale, slightly shortened from the original without compromising psychometric properties, has 10 items asking participants to rate how often they experienced feelings pertaining to stress on a scale of 0 (never) to 4 (very often). Total scores range from 0–40, with higher scores indicating a greater degree of perceived stress and lower scores indicating effective coping. The PSS was completed once at each assessment session.

#### 2.5.3. Visual Analogue Mood Scales

State mood was also assessed using the Bond and Lader [43] visual analogue mood scales, with additional “stress”, “fatigue” and “energy” visual analogue scales. These scales relate to immediate mood state (“right now”) and were used to assess mood and fatigue response to the completion of the SST functional imaging assessment. As such, visual analogue mood scales were administered at the start of the assessment visit and immediately following SST recordings.

The Bond and Lader mood scale consists of 16 items, each a 100-mm line with antonyms at either end (for example, “lethargic”-“energetic”). Three mood subscales are derived from these responses, as the average distance (millimetres) from the negative antonym of each contributing item, providing measures of “alertness”, “calmness” and “contentment”.

The additional visual analogue scales rated stress and fatigue along a 100-mm line, anchored at either end by “not at all” and “extremely”, where participants indicated current levels of being “stressed”, “mentally tired” and “physically tired”. For assessment of energy, participants rated their current level of “concentration”, “mental stamina” and “physical stamina” on a 100-mm line, anchored at either end by “very low” and “very high”. Across all visual analogue mood scales, higher scores indicate greater presence of the rated mood factor.

#### 2.5.4. State-Trait Anxiety Inventory

The State-Trait Anxiety Inventory (STAI) [44] contains a trait scale (STAI-T), which assesses the extent to which one is prone to anxiety as a stable personality characteristic, and also a state scale (STAI-S), which characterizes more transient experiences of anxiety “right now”. Each scale contains 20 items on which participants rated the subjective intensity of each short statement (“I am tense”) on a 4-point scale from 1 (not at all (STAI-S)/almost never (STAI-T)) to 4 (very much so (STAI-S)/almost always (STAI-T)). Scores on both scales range from 20–80, with higher scores indicating greater state or trait levels of anxiety. The STAI-T was administered a single time at the initial screening/familiarisation visit, whilst the STAI-S was administered at each assessment visit before any functional imaging in all participants. As with the visual analogue mood scales, participants undergoing SST assessment also underwent a second STAI-S measurement at the conclusion of these recordings to track the mood effects of this functional imaging assessment.

### 2.6. Data Analysis

Blood biochemical, mood and cognitive performance measures were analysed using the statistical software SPSS Version 23 (SPSS Inc., Chicago, IL, USA) for Windows. Data were first screened for the presence of outliers and skewed distributions prior to unblinding the dataset. After unblinding, where assumptions of the test were met, analysis of covariance (ANCOVA) examining results post-treatment controlling for baseline scores was conducted to assess the presence of a significant main effect of treatment as a between-group factor for each outcome measure, with a significance threshold *p* < 0.05. Where assumptions were not met, non-parametric alternatives were conducted, as described in relevant sections.

## 3. Results

Demographic data for the two treatment groups are provided in Table 2. Treatment groups did not significantly differ in age, Body Mass Index (BMI), years of education or trait anxiety. A count of treatment doses revealed that all participants satisfied the 80%–120% compliance criteria for inclusion in analysis.

**Table 2:** Demographic information for study participants (means (SD)). STAI, State-Trait Anxiety Inventory.

Variable	Multivitamin (*n* = 28)	Placebo (*n* = 30)
Male (*n*)	13	16
Female (*n*)	15	14
Age (years)	25.20 (5.33)	26.39 (4.42)
BMI (kg/m^2^)	23.42 (4.86)	24.52 (4.40)
Years of Education	16.63 (2.30)	16.04 (2.15)
Trait Anxiety (STAI-T)	34.29 (7.59)	35.53 (7.39)

### 3.1. Blood Biochemical Outcomes

Analysis of covariance was performed for haematological measures obtained at the follow-up visit controlling for baseline levels. Mean levels of blood B-vitamins, homocysteine and C-reactive protein are presented for the two treatment groups in Table 3 (note that the slightly reduced Ns for biomarker measures reflect that it was not possible to obtain both samples from some individuals, and the third party pathology laboratory was unable to analyse others). Significantly greater vitamin B6 levels were observed at follow-up for the MVM treatment group compared to placebo (*F*(1,41) = 50.08, *p* < 0.001, partial *η^2^* = 0.55). Due to evidence of violations of the statistical assumptions, the treatment effect on vitamin B6 levels was confirmed with a sensitivity analysis using non-parametric comparison of change from baseline to follow-up for this effect (*z* = −4.80, *p* < 0.001). Analyses performed on vitamin B12 levels found significant effects of MVM treatment compared to placebo on vitamin B12 (*F*(1,47) = 12.28, *p* = 0.001, partial *η^2^* = 0.21), with a non-significant trend towards a benefit of MVM treatment on red cell folate (*F*(1,45) = 3.11, *p* = 0.085). MVM treatment was also associated with significantly reduced homocysteine at follow-up when compared to placebo (*F*(1,43) = 7.35, *p* = 0.01, partial *η^2^* = 0.15). Analysis of covariance, examining C-reactive protein, glucose, lipids, electrolytes and indicators of liver and renal function levels at follow-up, controlling for baseline, found no significant treatment effect.

**Table 3:** Blood levels of B-vitamins, homocysteine and C-reactive protein for both treatment groups before and after four-weeks of treatment.

		Placebo			Multivitamin		
		Baseline	Follow-Up		Baseline	Follow-Up	
Measure (units)	*n*	*M (SD)*	*M (SD)*	*n*	*M (SD)*	*M (SD)*	*Sig*
Vit B6 (nmol/L)	22	85.00 (34.02)	89.73 (40.89)	22	84.95 (23.73)	251.45 (110.56)	***
Folate (nmol/L)	24	944.25 (190.09)	943.17 (205.81)	24	954.63 (188.58)	1022.71 (189.37)	†
Vit B12 (pmol/L)	24	304.88 (75.34)	301.04 (82.22)	26	286.15 (83.86)	350.58 (96.59)	***
Hcy (μmol/L)	22	11.43(2.24)	11.78 (1.81)	24	10.75 (3.57)	9.86 (3.23)	**
CRP (mg/L)	23	2.12 (3.75)	1.40 (1.56)	23	1.23 (2.28)	1.30 (1.66)	

Sig: Significance level: *** *p* ≤ 0.001; ** *p* ≤ 0.01; * *p* ≤ 0.05; ^†^ 0.05 < *p* < 0.10 (main effect of treatment). Measures: vitamin B6, red blood cell folate, vitamin B12, homocysteine, high sensitivity C-reactive protein.

### 3.2. Mood Outcomes

Measures of recent mood, assessed by POMS and PSS, and mood response to cognitive testing, assessed by visual analogue mood scales and STAI-S, are summarised for both baseline and follow-up assessment visits in Table 4 below. Treatment groups did not significantly differ at baseline on any measure of recent mood or mood response to the cognitive assessments.

#### 3.2.1. Recent Mood

**Figure 2:**
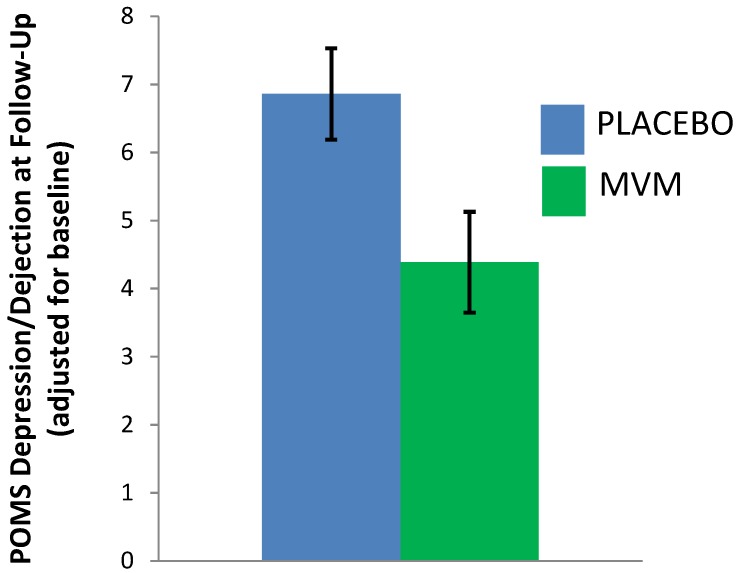
Post-treatment depression/dejection scores (POMS), adjusting for baseline (estimated marginal means, error bars ± 1 SE). MVM: Multivitamin/mineral treatment group.

An analysis of covariance, examining mood post-treatment, controlling for baseline, found that MVM treatment was associated with significantly lower scores on the depression/dejection subscale of the POMS, compared to placebo (*F*(1,48) = 5.96, *p* = 0.018, partial *η^2^* = 0.11; see Table 4 and Figure 2). There was no significant effect of treatment on other POMS subscales, nor total mood disturbance, nor the PSS (Table 4). Whilst underpowered for the exploration of treatment effects within gender, based on previous research in males demonstrating a positive effect of supplementation on PSS scores [26], this scale was probed for males separately. A non-significant trend towards lower PSS scores was observed after MVM supplementation (adjusted *M* = 11.41, SE = 1.17) compared to placebo (adjusted *M* = 14.12, SE = 1.14; *F*(1,27) = 2.76, *p* = 0.110, partial *η^2^* = 0.103).

Given the association between elevated homocysteine and depression [10], a “homocysteine hypothesis” of depression has been put forward [45]; however, recent intervention studies have failed to support a causal link between reducing homocysteine via B-vitamin supplementation and incidence of depression [46]. To explore the association between the significant reduction in subjective depression-dejection scores and reduced homocysteine within the MVM treatment group, a bivariate correlation between these two change scores was explored; however, no significant association was observed (*p* > 0.05).

**Table 4:** Measures of recent mood at baseline and follow-up for MVM and placebo treatment groups.

Recent Mood		*n*	Baseline	Follow-Up	ANCOVA	
			*M (SD)*	*M (SD)*	*F*	*p*
*Tension*	Placebo	27	7.59 (4.11)	7.96 (5.48)	0.01	0.913
	MVM	25	6.20 (4.18)	6.60 (5.44)		
*Confusion*	Placebo	28	7.43 (4.08)	7.18 (3.59)	0.55	0.462
	MVM	27	6.15 (4.77)	5.85 (4.70)		
*Vigour*	Placebo	28	20.00 (5.49)	17.21 (5.17)	2.85	0.097
	MVM	27	18.59 (6.88)	18.48 (6.96)		
*Anger*	Placebo	28	7.29 (5.45)	7.07 (5.81)	0.58	0.449
	MVM	26	5.23 (5.02)	4.77 (5.18)		
*Depression*	Placebo	28	7.14 (6.48)	7.39 (4.65)	5.96	0.018 *
	MVM	23	4.43 (5.49)	3.74 (3.98)		
*Fatigue*	Placebo	28	7.75 (4.44)	8.25 (4.55)	1.62	0.208
	MVM	27	6.52 (4.96)	6.30 (4.51)		
*TMD*	Placebo	26	13.50 (19.65)	19.69 (19.92)	1.56	0.218
	MVM	24	4.50 (19.92)	7.08 (24.72)		
*PSS*	Placebo	28	11.96 (5.07)	13.82 (6.28)	1.51	0.225
	MVM	27	12.96 (6.63)	12.85 (6.28)		

Recent mood measures: tension-anxiety, confusion-bewilderment, vigour-activity, anger-hostility, depression-dejection, fatigue-inertia and total mood disturbance of the Profile of Mood States (POMS), plus the Perceived Stress Scale. ANCOVA results correspond to the main effect of treatment on follow-up scores, controlling for baseline. MVM: Multivitamin/mineral treatment group.

#### 3.2.2. Mood Response to Cognitive Challenge

Completion of the cognitive tasks during SST had a significant effect on mood and fatigue at the baseline visit (Table 5). Across all participants at baseline, paired sample *t*-tests showed that completion of the cognitive testing during SST recordings resulted in a subjective decrease in mental stamina (*t*(32) = 3.60, *p* = 0.001), concentration (*t*(32) = 3.98, *p* < 0.001) and alertness (*t*(35) = 6.22, *p* < 0.001) while significantly increasing mental fatigue (*t*(38) = −3.51, *p* = 0.001).

For each state mood measure, an analysis of covariance was performed on the change in mood seen after completing the cognitive assessment with SST recordings at follow-up, controlling for the respective change as the baseline. Mood response to cognitive assessment did not significantly differ between treatment groups at follow-up, controlling for baseline (Table 5).

**Table 5:** Mood reactivity to cognitive assessment at baseline and follow-up for MVM and placebo treatment groups.

Mood Reactivity		*n*	Baseline	Follow-Up	ANCOVA	
			*M (SD)*	*M (SD)*	*F*	*p*
Alertness	Placebo	19	−11.13 (8.93)	−10.67 (10.19)	0.00	0.998
	MVM	17	−11.64 (13.17)	−10.79 (13.67)		
Calmness	Placebo	20	3.51 (15.86)	3.33 (16.63)	0.34	0.565
	MVM	19	−1.42 (15.63)	−0.90 (16.32)		
Contentedness	Placebo	19	0.70 (8.79)	4.05 (11.47)	0.64	0.428
	MVM	18	−1.80 (8.56)	−1.03 (14.79)		
Stress	Placebo	20	−4.95 (23.31)	−4.60 (23.49)	0.05	0.832
	MVM	19	−2.42 (16.32)	−4.68 (19.21)		
Fatigue	Placebo	20	21.20 (33.50)	12.70 (14.06)	0.49	0.490
	MVM	19	12.26 (25.87)	13.79 (30.16)		
Concentration	Placebo	18	−14.50 (16.31)	−10.94 (16.49)	0.01	0.932
	MVM	17	−12.35 (25.04)	−9.65 (24.68)		
Stamina	Placebo	17	−14.82 (14.80)	−9.65 (16.74)	0.80	0.379
	MVM	16	−6.06 (18.26)	−13.44 (22.51)		
STAI-S	Placebo	18	0.39 (5.37)	1.22 (3.25)	0.05	0.825
	MVM	19	0.79 (5.57)	1.05 (5.16)		

ANCOVA results correspond to the main effect of treatment on follow-up scores, controlling for baseline.

## 4. Discussion

The present study aimed to explore the effects of four weeks of MVM supplementation on mood and blood markers in healthy young adults (aged <40 years). The analysis of blood biochemical markers showed that active MVM treatment was associated with lowered homocysteine, in addition to increased vitamins B6 and B12, with a non-significant increase in red blood cell folate (B9). Compared to placebo, supplementation with MVM was also associated with significantly lower subjective reports of depression-dejection as measured using the Profile of Mood States. Cognitive challenge was associated with significant fatiguing effects; however, MVM treatment was not associated with any change in this response to cognitive assessment.

This report further highlights the potential positive mood effects of MVM supplementation in young healthy adults, in this case on the depression-dejection subscale of the POMS following 28 days of supplementation. A recent meta-analysis found evidence supporting the benefits to subjective stress and mood, including measures of depressed mood, in healthy adults, as a result of MVM supplementation [20]. The most common mood assessment outcomes in studies of healthy non-elderly adults have been the PSS and POMS, both included in the present study. We found no significant difference on the PSS, though a non-significant trend towards reduced scores on the PSS was noted in males. Amongst the mood dimensions of the POMS, only the depression-dejection subscale showed significant treatment-related differences. The lack of observed effects across other mood dimensions is possibly explained by a lack of power to detect relatively subtle changes in mood, as described in the meta-analysis of Long and Benton [20], whereby small-to-moderate effect sizes were observed for mood outcomes. This is perhaps best demonstrated with the “agreeable-hostile” dimension of the POMS, which was found to be significantly reduced by MVM supplementation in the meta-analysis [19], despite no single study reporting a significant treatment-related difference on this dimension.

In the current study, only one behavioural outcome was positively affected by MVM supplementation. No adjustment was made for multiple comparisons, though it is worth noting that the effects on depression-dejection are consistent with those previously reported in similar populations [20], with a *η^2^* = 0.11 translating to a Cohen’s f of 0.352, a medium-to-large effect size [47]. Whilst not wishing to over-interpret the data, we note that, of the ten behavioural outcome measures, nine were numerically improved in the MVM compared to the placebo group. It may be that the study was underpowered to detect significant findings in these measure (note that the study was powered on the neuroimaging component). Another possibility is that the effects of MVM supplementation are more readily detected in cohorts with sub-optimal nutrient status [48]. On the other hand, the cohort here did show positive shifts in blood vitamin status, suggesting that there may be physiological benefits from vitamin supplementation, even in non-deficient individuals. Furthermore, nutrient interventions may have more profound effects on psychiatric populations [49], so the effects reported here may be stronger in a clinical sample.

The lack of effects of MVM supplementation on cognitive challenge is unlike that reported by Haskell *et al*. [33]. There are, however, a number of methodological differences between the studies. These include differences in the nature of the challenge (multi-tasking *vs.* SST), duration of dosing (nine weeks *vs.* 28 days) and cohort (females aged 25–50 *vs.* both genders aged 18–39 years). These findings suggest that the nature of buffering against stress effects from MVM supplementation requires further characterization. 

Vitamins B6, B9 (folic acid) and B12, through their role as cofactors in the one-carbon metabolism pathway, are involved in the reduction of homocysteine to methionine and glutathione. The present study further confirms that MVM supplementation can reduce homocysteine levels in healthy young adults, even over a relatively short intervention period of four weeks. The extent to which homocysteine plays a causal role in negative health outcomes remains controversial, as the reduction of homocysteine through B-vitamin supplementation has failed to consistently demonstrate the health benefits implied by associations between homocysteine and cardiovascular disease [50], depressive conditions [46] and degenerative conditions [14]. The variability in response to these interventions may be at least partially mediated by genetic polymorphisms in the genes dictating the absorption, metabolism and function of B-vitamins *in vivo* [15]. In the present study, the MVM treatment-related reduction in homocysteine was not correlated with significant reductions in depression-dejection, suggesting a separate mechanism by which MVM treatment exerted positive mood effects. Kaplan *et al.* [51] proposed a series of potentially overlapping mechanisms by which MVM supplementation may exert positive effects on mood in the context of mood disorders, including compensating for genetic variability, which impacts on the efficiency with which micronutrients are used in metabolic processes (see also [15]), correcting deficient methylation processes, which may be caused by micronutrient deficiency or environmental influences, such as stress, which are required for the synthesis of neurotransmitters and other critical compounds, or epigenetic mechanisms, whereby MVM supplementation may reverse inadequate DNA methylation brought about by nutrient deficiency.

Whilst the present study further highlights potential positive mood effects of MVM supplementation in healthy adults, the study found no evidence of changes in cognitive performance on continuous performance or working memory tasks. It is important to note that these tasks were primarily implemented as activation tasks as part of functional brain activity assessments (to be reported elsewhere) and, as such, may not be maximally sensitive to detecting changes in cognitive performance. Indeed, previous studies to demonstrate cognitive performance changes in healthy young adults have utilised relatively extensive computerised cognitive assessment and/or multi-tasking batteries [26,33,34], suggesting validated, highly demanding assessments across a range of cognitive domains remain an important avenue for future research in order to fully characterise the effects of MVM supplementation in healthy adults [32].

## 5. Conclusions

The present study explored the effects of MVM supplementation in healthy younger adults, providing further support for potential mood benefits associated with MVM supplementation. Specifically, reductions in depressed/dejected mood were observed after MVM supplementation. In addition to increased blood levels of vitamins B6 and B12, analysis of blood biochemistry showed that MVM supplementation was associated with reduced homocysteine; however, these changes in homocysteine were not correlated with mood changes, suggesting a separate mechanism by which MVM supplementation impacted mood.
